# What Drives “Group Roaming”? A Study on the Pathway of “Digital Persuasion” in Media-Constructed Landscapes Behind Chinese Conformist Travel

**DOI:** 10.3390/bs15081056

**Published:** 2025-08-04

**Authors:** Chao Zhang, Di Jin, Jingwen Li

**Affiliations:** 1 School of Psychology, Henan University, North Section of Jinming Avenue, Kaifeng 475004, China; 2 School of Journalism and Communication, Henan University, No. 379 North Section of Mingli Road, Zhengzhou 450046, China; ljw1010@henu.edu.cn; 3 School of International Journalism and Communication, Beijing Foreign Studies University, No. 2 North Road, Xisanhuan, Beijing 100089, China; 202420321021@bfsu.edu.cn

**Keywords:** society of the spectacle, roaming, spectacle constraint, conformity-driven tourism, digital persuasion

## Abstract

In the era of digital intelligence, digital media landscapes increasingly influence cultural tourism consumption. Consumerism capitalizes on tourists’ superficial aesthetic commonalities, constructing a homogenized media imagination that leads to collective convergence in travel decisions, which obscures aspects of local culture, poses safety risks, and results in fleeting local tourism booms. In this study, semistructured interviews were conducted with 36 tourists, and NVivo12.0 was used for three-level node coding in a qualitative analysis to explore the digital media attributions of conformist travel behavior. The findings indicate that digital media landscapes exert a “digital persuasion” effect by reconstructing self-experience models, directing the individual gaze, and projecting idealized self-images. These mechanisms drive tourists to follow digital traffic trends and engage in imitative behaviors, ultimately shaping the phenomenon of “group roaming”, grounded in the psychological effect of herd behavior.

## 1. Introduction

With the development and penetration of digital media technologies, physical landscapes are digitally rendered into media spectacles, permeating the informational space of modern society in the form of virtual representations and subsequently shaping individuals’ travel intentions. Throughout various travel-related processes, such as information retrieval and consumer decision-making, individuals are finding it increasingly difficult to disentangle themselves from the embedded meanings of digital information, gradually becoming confined within algorithmically curated frameworks. As a result, holiday travel has shifted from a form of personalized leisure to an aesthetic labor of “pseudoindividuality.” In 2024, the number of domestic tourist trips in China reached 5.615 billion (Ministry of Culture and Tourism of the People’s Republic of China, 2025). Owing to the scenic representation in the game “Black Myth: Wukong”, Xinzhou city in Shanxi Province experienced a concentrated influx of tourists during the National Day holiday, with an average daily visitor volume of 390,500. China’s cultural and tourism consumption exhibits characteristics of significance, spatial clustering, and explosive growth (T. Zhang, 2024). Media symbols such as “Cozy southern snow goers” and “Prince and Princess” have become effective commercial instruments for amplifying niche cultural tourism, transforming previously marginal individualized wandering into a viral phenomenon across social media platforms and the cultural tourism sector. While digital media landscapes stimulate the growth of the tourism industry, they also present inherent drawbacks, including the obscuration of profound cultural essences and the hindrance of regional resource integration. Addressing the illusionary effects of digital landscape consumption and reconstructing emotional interactions between individuals and places has become essential.

### 1.1. A Study on Digital Media Landscapes and the Theory of Planned Behavior (TPB) Model

The media landscape constitutes a socially unified entity mediated through imagery (L. Liu, 2006). As a mimetic representation of social mobility, the digital media landscape synthesizes visual information into aggregated digital representations, occupying visual space and consequently shaping tourists’ latent perceptions and preconditioning their individual information choices (Yoon, 2019). The Theory of Planned Behavior (TPB), developed by Ajzen as an extension of the Theory of Reasoned Action (Ajzen, 1985), serves as a framework for explaining and predicting individuals’ behavioral intentions (S. Liu et al., 2025). This theory is employed to explain and predict individuals’ behavioral intentions and comprises five key influencing factors: attitude toward the behavior, subjective norm, perceived behavioral control, behavioral intention, and actual behavior (Ajzen, 2002). The attitude toward a behavior is primarily based on an individual’s beliefs about the outcomes of the behavior and the evaluation of those outcomes. The effects generated by digital media landscapes serve as a guiding force, influencing individuals’ behavioral attitudes (Kang & Han, 2015). Through platform algorithms, digital media landscapes cultivate audiences’ perceptions of subjective norms. Moreover, networked information sharing and “life-posting” behaviors within peer groups influence individuals’ behavioral intentions (Short et al., 2015). This process generates social or self-expectations, thereby altering individual intentions (Xiao et al., 2024). With the widespread diffusion of internet information in China, individual travel competence has become a fundamental form of behavioral literacy (L. Zhang et al., 2023). In addition to being influenced by close friends and family, individuals’ travel intentions are most strongly shaped by digital media landscapes (Sun et al., 2020). With the advent of China’s influencer economy and traffic-driven economy in the era of mass digitalization, electronic word-of-mouth (EWOM) has emerged as a perceptible external environment that facilitates communicative behaviors (Yin et al., 2024). This process gradually converts individual visual experiences into interactive practices within digital media landscapes, consequently leading to significant convergence in tourists’ perceptions, attitudes, behaviors, and habits (Wu, 2018). “Location tagging” and “spark a desire” behaviors serve as effective means for tourists to mitigate safety concerns, assess consumption levels, and reduce travel resistance, thereby reinforcing their behavioral intentions toward group-based decision-making. Among these factors, evaluations and shared experiences from same-age and same-gender groups on authoritative information platforms generate a peer effect, driving tourists to conform to the crowd through interactive support and competitive self-display (Y. Zhang & Xu, 2023) and engage in the social practices of self-presentation, self-perception, and identity construction.

Chinese commercial capital uses digital algorithms to selectively present users’ interaction traces and integrate them into the cultural landscape of the digital society. This, in turn, provides tourists with perceptual behaviors that have imitative value, prompting individuals to actively mimic the landscape’s value orientation, thereby generating a psychological impulse to visit firsthand (Best & Kellner, 2002). Conversely this individual longing driven by the landscape, on the one hand, leads tourists to engage in consumption and contribute to media production; on the other hand, it positions the individual as a potential “digital content curator,” further deepening the discourse of digital media landscapes through scenario setting and content reorganization (Corbett, 2011). In the “re-enchant” form of spiritual comfort provided by digital illusions, tourists forget the essential need to reach their authentic selves and instead shift their focus to emotional pleasure in virtual spaces. They actively invest their leisure time in visual consumption and aesthetic labor (J. Wang, 2023). Through the heightened beautification and deep condensation of regional cultural elements, this further conceals the consumerist nature of aesthetic labor. It constructs a “mediated otherness” that captivates the collective gaze of tourists, thereby triggering homogenized travel motivations (Zhou et al., 2023). Tourists aspire to independently curate their travel experiences but unconsciously become “Herd Behavior Tourists”, guided by the digital media landscape.

### 1.2. The Concept of “Group Roaming”: A New Model of Conformist Travel Under the Obscuration of the Media Landscape

“Roaming” originally referred to an unrestrained mode of wandering, free from the constraints of the media landscape. In city walks, tourists alleviate the “sense of placelessness” induced by modern urban life and reshape the human–place relationship through a culturally driven perceptual experience (Debo, 2006). As a journey from everyday life to novel experiences, travel signifies a crucial life trajectory for tourists within a social context (Fang, 2022). Tourists can perceive exoticized experiences and renew their own living spaces through personalized travel, temporarily escaping the constraints and anxieties of daily life. In this state of “relaxation,” they attain emotional compensation (Wu, 2014). However, in their pursuit of sensory gratification, tourists may inadvertently expose their latent desires, allowing algorithmic flows to tailor visual symbols accordingly. This process narrows their travel choices and limits their capacity for diverse perceptions. Digital media need only provide an “inverted” object of gaze—an aestheticized representation of an environment distinct from reality—to construct a “realistic image” that tourists are eager to follow. This, in turn, guides them toward deepening self-identification through visual consumption, leading to behaviors of imitation, emulation, and conformity (Dann, 1981). Personalized roaming is increasingly obscured by digital media landscapes, giving rise to the phenomenon of group “roaming,” which is characteristic of herd behavior.

Originally, travel was an embodied practice through which individuals reconstructed life encounters. Tourists often prefer destinations that significantly differ from their habitual environments, seeking to break routine constraints and fulfil their psychological desire for novel cultural experiences. However, in the digital era, the hyperreal sensations and perceptual gratifications provided by simulated contexts lead tourists to voluntarily integrate real-world consumption experiences into digital interactions. Within this digital space, the notion of “consumption as existence” proliferates, reinforcing a homogenized value system (Xiao et al., 2024). Consumerism is progressively intensified through contextual interactions, shaping tourism into a behavioral pathway for tourists to actualize their digital existence. In this sense, the image space replaces direct sensory experiences, emerging as a new arena where tourists seek consumption value and self-identity. As a result, individual travel experiences become stagnant and confined to performative acts such as “checking in” and purchasing, whereas deeper engagement with local culture, history, and humanistic insights is largely neglected.

### 1.3. Current Research

While existing studies have extensively explored the influence of digital culture on tourism intentions and behaviors, several critical limitations remain. First, current research perspectives tend to be skewed, as scholarly attention predominantly centers on the communicative effects of digital content while overlooking the proactive role of the digital media landscape as a mechanism of cultural production. Specifically, limited attention is paid to how digital landscapes construct tourist spaces and aesthetic imaginaries. Tourists are often positioned as passive recipients, with insufficient analysis of the dynamic interplay among behavioral intention, peer influence, and conformity psychology. Second, the theoretical depth of current scholarship remains underdeveloped. Discussions surrounding conformist tourism behaviors have largely remained at a descriptive level, lacking a cohesive analytical framework for the emerging phenomenon of “group roaming.” As tourists are increasingly subjected to the dual pressures of algorithmic logic and visual discipline, their decision-making logic becomes constrained—yet this behavioral shift has not been systematically theorized. Third, research in this field remains largely confined to the domains of tourism management and human geography, with limited integration of perspectives from communication studies and social psychology. As such, a cross-disciplinary analytical framework that incorporates both media logic and psychological mechanisms is urgently needed. By adopting an integrated perspective from communication and social psychology, this study seeks to systematically analyze how digital media landscapes embed themselves into, steer, and reshape contemporary Chinese tourists’ cognitive schemas and behavioral patterns. This approach not only contributes to the indigenization of key concepts such as the “society of the spectacle” and “media landscape” within China’s digital cultural context but also provides a solid theoretical and empirical foundation for understanding emerging patterns of conformity-based travel. This carries significant value for both theoretical advancement and practical guidance in an era where algorithmic mediation and aesthetic consumption increasingly define tourism experiences.

### 1.4. Theoretical Framework and Research Questions

This study integrates the Theory of the Society of the Spectacle with the Theory of Planned Behavior (TPB) and introduces the concept of “group roaming” based on the observed travel patterns shaped by conformity psychology. Distinct from individual travel decisions grounded in personal experiences, “group roaming” is essentially a manifestation of consumer convergence driven by commercial capital mediated by digital media landscapes. These landscapes synthesize fragmented elements of reality into directive symbols of consumption, enabling tourists to simultaneously express individuality and reduce decision-making pressure in the act of group roaming. However, the excessive saturation and aestheticization of landscape elements may solidify tourists’ cognitive dependencies, potentially overriding their genuine preferences and constraining emotional imagination and personal memory construction. Thus, “group roaming” also exemplifies the growing homogenization of tourist cognition under the dual pressures of visual content proliferation and the symbolic hegemony of consumption.

In terms of research scope, according to the China Online Audiovisual Development Research Report (China Netcasting Services Association, 2025), as of December 2024, China’s short-video user base had reached 1.04 billion, with a usage rate of 93.8%. The average daily usage time per person was 156 min—ranking first among all internet applications (Y. Liu, 2025). Therefore, this study focuses on the digital media landscapes shaped by China’s dominant short-video platforms, Douyin and WeChat Channels, analyzing how short-video characteristics and algorithmic mechanisms contribute to the construction of tourism imaginaries and the formation of behavioral intentions, thereby driving conformity in travel behaviors. Given the visual and video-driven focus of this research, platforms such as Xiaohongshu and Weibo, which are primarily based on image and text content, are not included in the current analysis. The study first focuses on the visual content and dissemination mechanisms presented by short-video platforms within the digital media landscape, aiming to reveal the pathways through which these elements influence Chinese tourists’ travel intentions. Second, it conducts an in-depth analysis of tourists’ subjective perceptions, emotional arousal, and behavioral logic during their engagement with digital media, clarifying the psychological motivations underlying the homogenization of travel behaviors. Finally, by integrating the Theory of Planned Behavior with the Theory of the Society of the Spectacle, the study constructs an analytical framework to interpret the digital persuasion mechanisms and conformity-driven travel selection pathways behind the phenomenon of “group wandering.” In doing so, the research addresses the core questions it sets out to explore: (1) In the context of consumerism, what are the core factors through which digital media landscapes influence the conformist travel intentions of Chinese tourists? (2) How do digital media landscapes obscure tourists’ autonomous perceptions and thereby achieve a form of implicit, cultivated persuasion?

## 2. Methods

### 2.1. Research Design

This study conforms with the Consolidated Criteria for Reporting Qualitative Research (COREQ) (Tong et al., 2007). The method of Interpretative Phenomenological Analysis (IPA) was adopted (Peat et al., 2019) for an in-depth understanding of how Chinese tourists, in a society saturated by digital media, form tourism-related cognition, construct behavioral pathways, and develop emotional identification with destinations under the influence of media content and platform mechanisms. This method emphasizes the subjective understanding of individuals’ lived experiences, focusing on how participants perceive and interpret the phenomena they encounter, in order to uncover the essential nature of those phenomena (Peat et al., 2019). Rather than merely describing observable behavior, this approach seeks to enter the internal perceptual world of media users, thereby revealing the underlying media logic and psychological mechanisms behind the phenomenon of “group roaming.” Guided by this methodology, this study employed in-depth interviews and textual analysis to explore how Chinese tourists developed conformity-based travel intentions within the short-video media landscape. This provided a qualitative foundation for modeling the digital persuasion of media landscapes and the selection paths of tourists’ conformity-based travel behavior.

### 2.2. Research Participants

This study employed a combination of purposeful sampling and snowball sampling to identify and select individuals who, under the influence of digital media, had relevant tourism experiences and were able to articulate their travel motivations, media perceptions, and behavioral logic. The inclusion criteria required participants to have traveled to popular domestic tourist destinations during public holidays and to have obtained travel-related information through short video content, which subsequently informed their actual travel behavior.

To avoid the limitations of single-case descriptions, data were collected across different geographic locations between April and November 2024. The research team conducted in-depth field interviews and participant observations in three major Chinese tourist cities, Kaifeng (Henan), Hangzhou (Zhejiang), and Zibo (Shandong), each of which had a peak single-day tourism reception exceeding 6 million visits. Participants were approached through various channels. These included on-site recruitment and participant observation in public spaces such as scenic areas and dining hubs; referrals from initial interviewees to acquaintances in their social networks, forming a snowball sampling chain; and introductions by key local actors, including cultural tourism staff, secondary school teachers, and Douyin influencers. This recruitment process ensured a structurally diverse sample in terms of occupation, age, and frequency of digital media usage. In-depth interviews were conducted, focusing on themes such as the digital media landscape in the digital age, symbolic meaning construction, and persuasive mechanisms. A total of 36 participants were recruited from 17 distinct social groups, including freelancers, industrial workers, volunteers, students, Douyin content creators, and high school teachers, ensuring a high level of social representativeness. Initially, 32 participants were selected as the core interview sample. Based on their demographic profiles, and following the principle of information saturation to enhance sample breadth and representativeness (Braun & Clarke, 2006), an additional 10 participants were considered for saturation verification. By the 4th added participant, no new thematic categories emerged, indicating saturation. The final sample size was thus determined to be 36 individuals. Among the 36 participants, 12 were recruited in Kaifeng, 11 in Hangzhou, and 13 in Zibo. Ages ranged from 21 to 62 years, with 17 male and 19 female participants. Participant demographics are presented in Table 1.

### 2.3. Data Collection

In-depth interviews were the primary method of data collection. In-depth interviews strike a balance between structured frameworks and flexibility, ensuring comprehensive coverage of key themes while allowing for the emergence of rich, qualitative data through insights into individual experiences (Dearnley, 2005; Kallio et al., 2016). Prior to data collection, three researchers collaboratively designed the interview guide based on the research questions, literature review, and theoretical framework. A pilot study was conducted to identify ambiguous or potentially misleading questions (In, 2017; Kunselman, 2024). After participants were fully informed about this study (including the researchers, objectives, and procedures) and signed the informed consent form, the second and third authors conducted the interviews. The second author, a male Ph.D. candidate, specializes in tourism behavior, media landscapes, and behavioral psychology in the context of digital media environments. He possesses strong theoretical grounding and research design capabilities. The third author, a female assistant researcher at the Center for Oral Communication and Culture Studies, Henan University, focuses on short-video platform communication and tourism consumption psychology, with substantial experience in conducting interviews. All interviews were conducted one-on-one, combining both online and offline formats, and were carried out in Chinese. Each interview lasted approximately 60 to 80 min. Follow-up interviews were conducted during the research process to explore new findings and refine emerging themes. In total, approximately 517,000 Chinese characters across interview transcripts and observation notes were compiled. With participants’ explicit consent, all interviews were audio-recorded in their entirety. To ensure data integrity and security, all research materials were systematically digitized and securely managed. Audio files and transcribed texts were stored on encrypted storage devices to prevent data breaches or loss.

### 2.4. Data Analysis

After completing 36 interviews, the research team confirmed data saturation and subsequently proceeded to the data analysis stage. The analysis employed the three-level node coding method using NVivo 12.0 Plus, aiming to construct a clear and hierarchical coding structure. First, the second and third authors imported the audio recordings—obtained with informed consent—into an intelligent transcription system to generate textual transcripts. They then verified the consistency between the transcripts and the original recordings. All transcripts were subsequently translated from Chinese to English. Each participant was assigned a de-identified code, and the entire transcription and translation process was conducted under the oversight of all three authors. Next, the transcripts were imported into NVivo 12.0 Plus for initial data organization using word frequency analysis, synonym matching, and automatic node generation. The second and third authors independently coded the transcripts line by line, following an open coding approach. They then compared their coding outcomes, discussed agreements and discrepancies, and merged or refined codes where necessary. In the third phase, the second author generated preliminary themes based on the coding results and aligned them with the pre-established theoretical framework. The first author then reviewed the thematic structure to ensure adequate coverage of the data and suggested revisions or additions for any ambiguous or incomplete themes. Finally, all three authors jointly reviewed the final themes, confirming their alignment with the dataset and the research questions. They checked for overlapping or redundant themes, merged them where appropriate, and finalized theme names along with representative key data excerpts to support each theme. To ensure the reliability and validity of the coding process, several analytical techniques were applied, including constant comparison, content analysis, reliability measurement, and saturation testing. The inter-coder reliability for content analysis reached R = 0.92, indicating a high level of consistency among the primary coder’s classifications. Therefore, the primary coder’s results were deemed valid for use in further analysis.

## 3. Results

**Figure 1:**
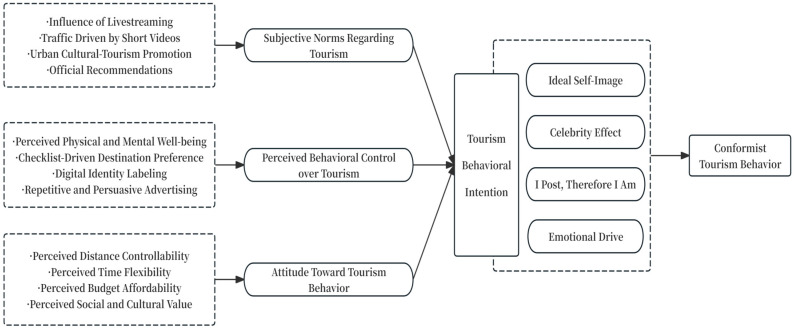
The pathway of digital persuasion via media landscapes and tourists’ conformist travel decisions.

Consumers are essentially “identity seekers,” and tourists construct their identity through consumption behaviors, thereby gaining a sense of self-realization and self-achievement (Giddens, 1992). In order to sustain and elevate their sensory gratification, individuals increasingly suppress individual reflection and instead collectively engage in landscape consumption. Within this process of shared consumption, they pursue self-aestheticization and public presentation, which transforms tourism into a form of aesthetic labor regulated by digital visual culture. The findings of this study are categorized into two main sections: (1) the key factors through which digital media landscapes shape Chinese tourists’ conformist travel intentions; (2) the pathways by which digital media landscapes obscure tourists’ autonomous perceptions, thereby achieving cultivation-based persuasion. Detailed findings are presented in Table 2 and Figure 1.

### 3.1. Components of Travel Behavioral Intention

Drawing on the Theory of Planned Behavior (TPB) and utilizing NVivo 12.0 Plus for qualitative data analysis, interview transcripts from 36 tourists underwent three-level node coding. The analysis identified one first-level node—Travel Behavioral Intention and three second-level nodes—Subjective Norms Regarding Tourism, Perceived Behavioral Control over Tourism, and Attitude Toward Tourism Behavior—along with their corresponding coding frequencies and coverage rates. These nodes collectively represent the core factors influencing the conformist travel behavioral intentions of Chinese tourists. The number of reference points reflects the frequency of each node’s appearance in the original textual data, while the coverage rate indicates the proportion of text occupied by each coded node. Detailed results are presented in Table 2.

#### 3.1.1. Subjective Norms Regarding Tourism

Within the framework of the Theory of Planned Behavior (TPB), subjective norms refer to the perceived social pressure from others regarding whether one should engage in a specific behavior (Ajzen, 2011). In this study, Subjective Norms of Travel Behavior manifested in tourists’ perceptions of prevailing travel trends and behavioral paradigms as presented through short videos, livestreams, and discourse on social media platforms. These perceptions were subsequently internalized and translated into individual behavioral intentions. According to the NVivo three-level coding results, a total of 351 reference points were identified under this node, with the majority falling into four key categories: influence of livestreaming (78), traffic driven by short videos (93), urban cultural-tourism promotion (85), and official recommendations (95). Digital media production tends to actively cater to audiences’ emotional desires, propelling the cultural-tourism industry toward an entertainment- and consumer-oriented mode. As vision is a primary channel for perceiving information, it facilitates tourists’ multisensory engagement with travel experiences. The resulting visual centrism contributes to the transformation of modern society into a “society of the spectacle.” In this context, the digital sphere becomes an “imagined world” in the visual sense—one imbued with performative, transmissible, and self-replicating visual elements. These coalesce into what may be described as a “media dome,” shaping the widely circulated imaginary of “places one ought to visit.” Such visualized persuasion subtly drives individuals to identify with, and conform to, collective travel decisions. As one interviewee noted, *“Sometimes, after watching too many of those videos, it really feels like I’m being pushed along—especially those vlog-style clips set to music. They’re hard to resist.”* (DZY20240425) The visual experience promoted by short-video platforms reinforces tourists’ homogenized perceptions of popular landmarks and trending destinations, cultivating a normative atmosphere of group intent. At the same time, official endorsements and municipal promotional content—backed by institutional authority and policy—further enhance tourists’ sense of safety and trust in certain destinations. As another interviewee stated, *“Before going on trips, my parents always watch travel recommendation programs on CCTV. They feel those places are ‘reliable’ and less likely to disappoint.”* (ZMY20240423) Under the collective cues of “all major platforms are recommending it” and “everyone else is going,” tourists begin to internalize the mediated consensus as personal intention. This reflects a behavioral logic of adaptive conformity, highlighting the influential role of digital media in shaping subjective norms of travel behavior.

#### 3.1.2. Perceived Behavioral Control over Tourism

Perceived behavioral control refers to an individual’s assessment of the availability of resources, capabilities, and opportunities necessary to perform a specific behavior (Suntornsan et al., 2022). In the context of this study, Perceived Behavioral Control over Travel Decisions can be understood as tourists’ holistic evaluation of their ability to access and utilize external media resources, such as platform-guided itineraries, ranking lists, and symbolic visual cues, offered by digital platforms. A total of 386 coded references were identified under this node, focusing primarily on four thematic dimensions: perceived physical and mental well-being (102), checklist-driven destination preference (107), digital identity labeling (83), and repetitive and persuasive advertising (94). In order to cater to collective preferences, capital-driven algorithms elevate visual elements from shareable signs into seductive, media-optimized symbols—designed for social media virality. This transformation satisfies tourists’ growing desire for unique experiences and enhanced value-based consumption (Y. Liu & Jiang, 2021). Under the sway of digital traffic, media landscapes evolve into obscuring discursive mechanisms that substitute lived experiences as the primary guide for consumption choices. As a result, travel behavior becomes a form of replicable aesthetic labor, serving to perpetuate the media spectacle and attract new participants into the consumption fold. As one interviewee stated, *“I planned my entire itinerary based on short videos—someone had already done it and filmed it, so it felt safe to follow.”* (XLJ20240503) “Checklist-style” short videos reduce the cost of planning while enhancing individuals’ confidence in the success of their travel experiences. Visual symbols such as “healing landmarks” and “relaxation-themed routes” serve as emotionally resonant ranking content, offering tourists a sense of spatial reliability and reinforcing the connection between travel and psychological comfort. These representations also encourage tourists to share their experiences in virtual spaces, further drawing upon others’ narratives to shape their own travel plans (Sandbye, 2014). The emergence of “digital identity labeling” adds another layer of symbolic significance, allowing individuals to frame their travels as expressions of a meaningful lifestyle, *“Going to a place isn’t just about having fun—it’s like tagging myself with an ‘interesting life.’”* (LJX20240502) Moreover, the repeated push notifications and content loops on short-video platforms invite users to compare their real lives with idealized media portrayals. Through this comparison, tourists incrementally absorb the visual logic advocated by digital media landscapes, ultimately fostering behavioral intention to travel.

#### 3.1.3. Attitude Toward Tourism Behavior

Attitude toward behavior refers to an individual’s overall evaluation of performing a particular action, encompassing both their cognitive assessment of expected outcomes and their emotional predispositions (Ajzen, 1985). In this study, Attitudes Toward Travel Behavior are understood as tourists’ comprehensive evaluations formed through value judgments, experiential expectations, and meaning-making processes surrounding travel. These attitudes are jointly influenced by the structure of information, the narrative construction of media, and individual emotional mobilization. Based on the third-level node coding conducted in NVivo 12.0 Plus, 436 coded references relevant to travel behavior attitudes were found. These primarily fell into four thematic third-level nodes: perceived distance controllability (105), perceived time flexibility (112), perceived budget affordability (128), and perceived social and cultural value (91). The high number of references to budget and time considerations, both exceeding 110, suggests that tourists widely view these as foundational conditions for executing travel plans. As one participant remarked, *“I dislike trips that require heavy spending and tight schedules—it’s exhausting and meaningless. Ideally, I want a trip that I can complete within a day, with minimal cost and maximum relaxation.”* (JXX20240503) Mediated visual behavior, condensed and amplified by algorithmic technologies, becomes a form of collective aesthetic labor within “information cocoons,” where digital content circulates in spectacular forms. These visual representations, through algorithmically constructed echo chambers, reinforce homogenous decision-making among potential travelers. As another participant noted, *“I kept seeing videos of West Lake, especially influencers reenacting scenes from The Legend of the White Snake. I finally decided to go. But during the National Day holiday, it was so crowded I got pushed onto the Broken Bridge—there was no actual experience. Honestly, walking with friends through the alleys of Hangzhou on a City Walk felt more authentic.”* (YCY20240428) Data, as a unique symbolic form, lend itself to repeated validation and confirmation. Their empirical clarity and interpretability lend digital media landscapes a sense of credibility, enhancing tourists’ perceived feasibility of travel behaviors. *“Every time our family plans a trip, we rely on what’s trending online. We check Douyin to see what’s fun, and then save up vacation days to go. But once we arrive, it’s the same kind of commercial streets and snacks everywhere. If you don’t dive into the local culture, it’s really no different from staying home for a staycation.”* (LTY20240424) By promoting data-based recommendations, the media landscape can temporarily alleviate individuals’ decision-making anxiety. However, this also results in the erosion of autonomous decoding ability, leaving some tourists vulnerable to digital influence and detaching emotional fulfillment from personal experience. Visual experiences, as vehicles of semiotic power, are echoed and reinforced in digital environments, reconfiguring collective perception and shaping consumption intentions. While travel ought to be characterized by the unpredictability, multiplicity, and spontaneity inherent in postmodern experiences, it is often subsumed into the logic of consumerism through aestheticized visual presentation. Even before arriving at a destination, many tourists project themselves into the digital realm, willingly becoming loyal consumers of visual content in pursuit of identity enhancement and self-validation.

#### 3.1.4. Gendered Orientation in Conformity-Based Travel Behavior

The analysis of in-depth interviews with 36 participants revealed that, although the sample was demographically diverse, factors such as occupation, educational background, and age were not the key determinants influencing travel behavioral intention. Rather, gender differences emerged as one of the major factors shaping conformity-based travel behavior under the influence of the digital media landscape. Previous research has demonstrated that women tend to spend more time online each week and exhibit more favorable attitudes toward both online and offline sources of travel information (Kim et al., 2007). In addition, the 2024 Annual Female Travel Consumption Report released by Tuniu, a leading Chinese travel app, indicates that women account for nearly 60% of all tourist trips, making them the dominant force in the tourism market. The report highlights that women often hold primary decision-making power, especially in family travel contexts, where they typically act as gatekeepers—initiating travel ideas, gathering information, and planning itineraries. Moreover, the influence of social media and digital platforms on women’s travel decisions has become increasingly prominent (China Tourism Association, 2025).

Female tourists demonstrate higher levels of agency and engagement in media perception, information acquisition, and emotional responsiveness. They are more susceptible to emotional mobilization through short videos and livestream scenes, and they exhibit stronger identification with and anticipation of the aesthetic scenarios constructed by media content. Among the 19 female participants, 15 explicitly stated that they were prompted to travel due to emotionally resonant video content or atmosphere construction. The age range of these 15 women spanned from 23 to 40 years old. For instance, one participant noted, *“The jazz music background in a city street-style video instantly hit me, it captured exactly the lifestyle I longed for.”* (ZSP20240415) A total of 13 women clearly stated that the sense of ambience and aesthetic imagery in short videos directly sparked their desire for immediate travel. *“I just felt that city was made for me—it felt so relaxed, so I bought a ticket and went.”* (CS20240501) Women are more likely to be “influenced” by short videos, viewing tourism as a pathway for healing, emotional release, and self-affirmation. They tend to make travel decisions based on visual content and shape consumer trajectories grounded in self-identification. In contrast, among the 17 male participants, 11 indicated that they focused more on practicality and planning in travel content and openly admitted that most travel decisions were made in response to suggestions initiated by women in their household. As one male participant stated, *“Where we travel is based on what my wife sees on her feed. If she wants to go, I go.”* (ZMY20240423) Another added, *“Honestly, I don’t really watch travel content. But if she sees something she thinks suits us, I’ll just go along.”* (SR20240425) These findings reveal that gender differences are evident not only in how individuals perceive media content but also in decision-making authority and degree of media reliance. Female tourists are more readily socialized into the digital media landscape and tend to act as “travel initiators” within family settings, becoming key drivers of group-based travel behavior.

### 3.2. Key Elements of Travel Behavioral Intention

Within the framework of the Theory of Planned Behavior (TPB), behavioral intention is regarded as the immediate driving force behind the execution of a particular behavior. Under the influence of the digital media landscape, tourists’ travel behavioral intentions exhibit distinct characteristics. To further clarify the composition of such behavioral intentions, this study, building upon the TPB model and drawing on in-depth interviews with 36 participants, identified four major motivational categories underlying travel intentions: ideal self-image construction, celebrity effect, “I post, therefore I am”, and emotional drive. These four types of behavioral intention all stem from prolonged exposure to and deep engagement with content in the digital media landscape. They reflect the dynamic interweaving of multiple travel motivations among tourists. As direct analytical outcomes derived from interview data, these categories enhance the explanatory power of the TPB in the context of digital media, offering a more nuanced psychological portrait of the evolution from behavioral intention to conformity-based travel behavior. They also provide a more persuasive framework for understanding how digital media influences such collective travel decisions.

#### 3.2.1. Ideal Self-Image Construction

Among the 36 participants, 28 explicitly stated that their decision to travel to a particular destination was motivated by having “seen the person they wished to become” through online video content. In their view, travel is not merely a spatial displacement but also a transformation of roles and a reconstruction of one’s persona. By producing positive imagery, the media represents travel behavior in a way that guides individuals to shift their travel perspective from self-directed experience to imitation and even the transcendence of “others” in trending destinations. Compared to autonomous exploration and personalized presentation, crafting an ideal self-image via the digital media landscape is perceived by some tourists as more efficient. This gives rise to conformity-based behaviors such as trend-following check-ins and influencer-driven consumption (Cheng & Gao, 2021). Originally intended as a relief from the fast pace of daily life, travel decisions—under the acceleration of information flow—have transformed into moments of performative urgency. Travel consumption thus becomes a means of seeking recognition through self-presentation, leading to the alienation of the playful essence of travel within the logic of social identity groupings imposed by the media landscape. *“I’m under a lot of pressure at work. When I saw a blogger drinking tea and watching flowers in a village, I thought—that’s who I want to be. That life looked so beautiful. I booked a flight to the exact place she filmed. I even bought the same clothes and filmed the same shots. I know that wasn’t the real me, but in that moment, I felt like I was living as the person I wanted to become.”* (LJX20240502) In their pursuit of layered recognition across both physical and virtual spaces, tourists exhibit increasing homogeneity in their emotional, consumer, and discursive behaviors. This reconfigures the interaction between people and places, and between self and society, within the idealized aesthetics of the digital landscape. *“My colleagues and friends on social media always post during holidays: where they went, what they ate, what exhibitions they saw. I didn’t really have anywhere I wanted to go, but I felt like if I posted nothing, I’d fall behind. So I picked a city trending online. I didn’t plan anything myself—just searched for a ‘2-day viral itinerary’ on the platform. It didn’t feel like traveling; more like submitting proof of life.”* (DD20240425) Through building social sharing mechanisms and enriching the ritualized meaning of participation, the digital media landscape fabricates a false sense of collective equality, masking the anxiety and tension resulting from the dissolution of real-life identities in virtual space. In constructing their self-image, tourists imitate symbolic occupation practices of virtual personas and transplant these behavioral models into real-life value systems in hopes of reclaiming and reconstructing their tangible self-identity.

#### 3.2.2. Celebrity Effect as Behavioral Driver

The digital media landscape fabricates symbolic labels such as “influencer” and “top-tier creator,” imbuing them with idolized social significance. Within the framework of fan-based economies, this leads tourists to gradually shift away from cultural curiosity and toward travel imaginations shaped by symbolic rewards embedded in visualized landscapes. Among the 36 interviewees, 27 clearly acknowledged that their travel destination choices were influenced by internet celebrities or travel bloggers. Specifically, 17 participants stated that they followed the itineraries of specific influencers, while 10 mentioned traveling to locations simply because a favorite celebrity or video creator had visited them. This indicates that the celebrity effect has evolved in the digital space, from the mere dissemination of content to a substantial guiding force on user behavior, becoming a crucial psychological driver in the formation of travel intentions. *“If my idol has been there, of course I want to go too. Isn’t checking in at the same place a way to prove I also left a mark on this world?”* (NZY20240502) Rather than empowering individuals through technological affordances, such travel practices mediated by digital platforms reflect a process of self-commodification (Abidin, 2016). Within the “dream-making space” of the digital media landscape, individuals engage in spatial exploration and image capture, often unaware that their sense of self is gradually subsumed into a glossy, preconfigured virtual narrative. They become commodified spectacle elements within the digital media’s visual economy (Jin et al., 2023). As Debord noted, “The more he contemplates, the less he lives (Y. Zhang, 2006).” Once these mediated images are internalized as personal desires, individuals forfeit the authenticity of their own needs, becoming detached from what their inner selves truly seek. *“I checked in at Lingyin Temple just to make a Douyin short video to ‘respond’ to my idol. I needed to prove I’d been there too, and I wanted to shoot it even better than anyone else.”* (MXY20241001) By constructing diverse “traffic labels,” visual media precisely captures the tourist’s aspiration to reframe their identity and escape mundane routines. However, despite seeking personalized fulfillment, tourists are absorbed into the logic of consumer collectivism and visual conformity. In this process, they become producers of symbols, reinforcing the stock of digital imagery and further entrenching themselves in the aesthetic regime of the spectacle. The interplay between celebrity influence and digital landscape renders tourists not only consumers of mediated experiences but also performers within a pre-scripted visual economy.

#### 3.2.3. I Post, Therefore I Am

As digital media landscapes become increasingly embedded in tourism practices, posting has evolved into a form of visual representation that fulfills a function of social recognition. All participants reported sharing images or videos via short-video platforms or social networks during or after their trips to document their journeys, display their status, or respond to the expectations of others. Among them, 19 female and 12 male respondents explicitly stated that posting was not merely an act of sharing but had become an indispensable part of the travel experience. *“If I don’t post anything, it’s like the trip never really happened.”* (DTT20240425) *“I have many friends on Douyin; sharing posts helps record where I’ve been while also updating others on my current state.”* (GGM20240426) For some travelers, the photo-worthiness of a destination has become a key selection criterion. Five female participants mentioned that, before setting out, they actively searched on platforms such as Rednote or Bilibili for destinations with a “high photo output rate,” including visual composition tips, to plan their shooting routes accordingly. *“I plan my travel routes just for photos. If I can’t take good pictures, I might not even go—because without photographic proof, it feels like the trip never happened.”* (NZY20240502) Tourists present a coherent identity narrative through social media, using the aesthetics of the landscape to affirm their self-image. The digital media landscape, as a highly credible “intermediary,” encourages tourists to rely on its discursive guidance, thus shifting their self-presentation behavior into the evaluation system of the digital media landscape. *“Knowing that this spot is crowded, who would want to follow the trend? However, the whole family only has time during holidays, and there are only one or two chances per year. What if we pick an unpopular spot, and it turns out to be the wrong choice? Well, let us just squeeze in. There must be a reason why so many people go, and it still feels lively.”* (LTY20240424) As the German aesthetician Heinz Petswald stated, there is a paradox in urban walking: “It is highly purposeful, yet it is integrated into a structure that leads to a destination without any specific purpose (Petswald & Deng, 2013).” When individuals embark on a goal-oriented trip to relieve stress or seek healing, can this activity still maintain its behavioral purposelessness? In the face of the unknown in a “foreign place,” it becomes difficult for people not to “seek benefits and avoid harm,” and media information thus becomes the optimal solution to avoid “missteps” and “detours.” At this point, commercial capital, to highlight its empirical nature and value scale, shapes itself through the digital media landscape as an authoritative and universal solution, thereby drawing “digital spectators” into the boundaries of the landscape.

#### 3.2.4. Emotional Drive

Through the contextualized aggregation of scattered visual symbols, the digital media landscape effectively integrates the inner aspirations of tourists, transforming their shared psychological desires into visually accessible experiences that can be previewed, thus triggering sensory connections and psychological simulations. In this landscape–driven synesthetic effect, individuals’ travel decisions are deeply influenced by the digital “filter,” shifting toward embodied practices within the landscape’s preset framework. Crowd tourism behavior thus becomes a real-world reflection of the “visual–emotional” consumption relationship in the digital space, where producers, using digital visual symbols as intermediaries, establish hidden associations with individual consumer practices. By crafting highly personalized visual content and reinforcing immersive narrative contexts, digital media landscapes often trigger emotional responses in tourists before any rational evaluation occurs. When such landscapes present idealized lifestyles or picturesque moments, they not only satisfy aesthetic expectations but also stimulate experiential motivations. Among the 36 participants in this study, 18 indicated that being emotionally “moved” was a major factor in their travel decisions—more so than the practical value of the information. Notably, when a travel destination resonates with one’s personal emotional network—such as being a place where friends or family reside—the emotional appeal of the digital media landscape is significantly amplified. Twelve participants mentioned that they decided to travel to cities frequently featured in viral videos because they also happened to be locations where acquaintances lived, effectively integrating visiting friends and reliving shared memories into a legitimate travel motive. For example, one participant shared, *“I saw a short video about how great the barbecue is in Zibo. My college roommate lives there, so I immediately wanted to go—it was also a way to relive our university days.”* (GLS20240417) Another said, *“After seeing so many videos about Hangzhou, I became really interested. My brother works there, so I thought we could hang out together.”* (SR20240425) Additionally, eight female interviewees noted that cities labeled with emotional keywords such as “healing,” “soothing,” or “relaxing” on digital platforms were especially appealing when a familiar person happened to live there. *“My friend lives in the place Douyin calls ‘a dream of the Song Dynasty.’ I booked a ticket right after watching the video.”* (WCY20240430) In such cases, the emotional resonance of the digital landscape combines with real-life social ties to lend immediacy and legitimacy to travel decisions. However, while this emotion-driven travel intention may appear to reflect autonomous choice, it is in fact often shaped and activated by platform-guided “emotional compliance behaviors.” As short video algorithms increasingly highlight elements such as “friends are here” and “good vibes,” users’ original emotional motives risk being excessively symbolized. This symbolic overcoding compresses the space for genuine cultural exploration tailored to individual needs, drawing tourists deeper into the platform-constructed order of the spectacle and weakening their intrinsic desire for self-directed discovery. Ultimately, emotional drivers may slide into emotional homogenization.

## 4. Discussion

In the digital age, online information has become a “guide” that directs tourists in pursuing an “expansive experience” in real spaces, carries the value consensus of consumerism, and continually influences individuals’ personal choices. Capital, through the construction of hyperreal narrative spaces, creates information cocoons that standardize the decision-making orientations of tourists after their “thought processes,” thereby encouraging them to voluntarily join the conformist crowd.

### 4.1. Constructing the Complete Pathway of Persuasion in Digital Media Landscapes

With the penetration of digital visual technology into the private domains of individuals, the production of mass imagination can no longer meet the demands of capital accumulation. Therefore, commercial capital makes full use of data algorithm technologies, treating audience behaviors such as likes, collections, and browsing durations as analytical samples. This enables the precise capture of audience expectations, refinement of production models, and framing of consumption trends. The media landscape it has constructed has gradually surpassed the influence of mainstream mass media on individual travel, with digital media landscapes superficially offering tourists labels of freedom. In reality, the experiential model has long been predefined, leading to a convergence of travel choices despite different paths. The visual, intuitive images of most tourists gradually replaced independent thinking, transferring the “custodianship” and “definition” of individuality to the creators of the digital media landscape. These creators, in turn, guide audience consumption by beautifying the images of places. The media landscape, detached from reality, enhances its intangible control over tourists through widespread entertainment, thereby eroding their critical and creative abilities. It subtly and covertly persuades tourists to accept and rely on conformist choices.

As a spectacle experience, travel involves cultural customs, embodied memories, and emotional enjoyment, allowing tourists to achieve a multicultural reconstruction of their self-identity through the life modes of “other places” and “others.” The digital media landscape shifts this path of real-world cultural construction into a simulated space, making travel decisions a product of the interplay between the discourse of the landscape and individual demands. Commercial capital, through traffic rewards, drives the interactive desires of tourists, causing individuals to autonomously obscure the essence of mass tourism as aesthetic labor in their pursuit of identity fulfilment, thereby constructing a causal relationship between consumer practices and identity recognition in the minds of tourists. The persuasion in travel formed by digital media landscapes is characterized by potentiality, concealment, and guidance. Individuals live within the media environment, experiencing a sense of “being deep in the clouds and unaware of the distance, yet only within this mountain,” thereby being subtly influenced and shaped by the digital media landscape when making decisions (Qiang & Zhang, 2023). As an external data cluster for tourists to receive travel information, the digital media landscape further refines individuals’ aspirations for the “ideal life” through traffic algorithms. It then transforms these aspirations into easily imitable simulated experiences, thereby guiding tourists into the digital media landscape and constructing a convergent travel experience model. The visualization and easily replicable simulated scenarios compel tourists to flock into the landscape in search of recognition, causing cultural tourism consumption driven by traffic algorithms to gradually evolve into group behavior influenced by the emotions of the digital space.

Therefore, in the process of making travel decisions, tourists do not simply arrive at a real-world “other place,” but rather at a coupled space formed by the interplay of individual expectations, landscape influence, and self-concealment. The individual behaviors within the space are constrained and shaped by algorithms, ultimately transforming into the “aesthetic” aspect of the situational material, further embellishing the real construction meaning of mass tourism. Consumerism creates a false illusion of freedom in tourism consumption, concealing the nihilistic nature of landscape consumption and constructing the public’s emotional expectations and cultural aspirations toward digital media landscapes. This, in turn, drives tourists to autonomously enter the virtual framing of mediated landscapes in pursuit of the mirrored expectation of their “true self.” Tourists may believe that through digital visual consumption activities, they are showcasing their individuality and enhancing the external bodily perception and emotional relaxation caused by travel. In reality, however, they shape the “other’s” landscape, reinforcing or filling the information echo chamber of the destination’s digital media landscape.

### 4.2. The Reconstruction of Individual Meaning and Identity in the Context of Media Landscapes

A constructed situation refers to a carefully curated moment of life within a unified environment (Y. Liu, 2010). The emphasis is on tourists redesigning and experiencing the value of life on the basis of their authentic desires, with the aim of constructing personal living spaces and urban public spaces rooted in the liberation of free will (G. Wang, 2013). Roaming, as a constructed situation of daily life, is currently influenced by digital media, becoming a phenomenon shaped by the interaction of alienated needs and media dependence. It subsequently reflects issues such as the loss of authentic experience and the ossification of individual meaning. Constructing critical media literacy as a key mechanism to help tourists achieve self-liberation can effectively eliminate redundant information, optimize the construction of information ethics and legal frameworks, and enable tourists to regain individual cognition and autonomous critical thinking skills. This, in turn, helps tourists reconnect with local cultural knowledge and, through “deep tourism,” achieve personalized decoding of the symbols of “other places.”

The “dialectical disengagement from spectacular discourse” of landscape discourse by tourists is not a unilateral rejection of media information but a dialectical examination of the attributes of media tools from a humanistic perspective. It is self-driven reclaiming based on an individual’s deep media awareness and personal needs. We need to be vigilant about the intrusion of digital media landscapes into the space of individual needs. However, we should also recognize the positive effects of commercial marketing and media guidance. The digital media landscapes they construct still hold value in enhancing the social value of roaming and promoting personalized travel ideas. In the digital age, media has become an inseparable cultural skin that coexists with individuals. Commercial marketing and media staging can easily lead individuals to experience a sense of identity displacement amid “loss and adventure.” The key to mitigating this identity risk lies in cultivating tourists’ local cultural literacy and replacing the psychological persuasion of media landscapes with offline experiential learning. This approach enables tourists to reconstruct the rational meaning of landscapes through autonomous interpretation, allowing them to perceive deeper cultural spaces through self-fulfilling experiences. The reconfiguration of value in physical space can effectively liberate tourists from the constraints of commodified bodily roles, allowing them to break free from the visual order of digital media landscapes. This process enables tourists to reclaim the cultural significance of real-world social circles, deeply embed their bodily perceptions within local cultures, and construct a personalized “tourism perspective” rooted in diverse cultural identities.

## 5. Conclusions

The findings of this study indicate that subjective travel norms, perceived behavioral control, and travel attitudes are key factors driving conformity-based travel behavior within the digital media landscape. Moreover, short-video platforms shape idealized personas, create celebrity effects, evoke emotional resonance, and foster social identification, thereby gradually obscuring tourists’ original perceptions and autonomous judgment. This process constitutes an enculturative pathway of “digital persuasion.” The study explicitly reveals the mechanisms through which the digital media landscape influences Chinese tourists to engage in conformity-based travel behavior, along with the underlying psychological motivations, thereby fulfilling the established research objectives. In the visually dominant digital media space, the spectacle establishes a comprehensive and intricate psychological intervention mechanism, transitioning from self-presentation to consumer guidance. This process erodes the boundary between leisure and labor, transforming “roaming” from an act of personal relaxation into aesthetic labor, ultimately manifesting as “group roaming” characterized by conformity. The travel discourse constructed by media platforms embeds a latent chain of meaning that assimilates group consumption, further reinforcing the influence and control of the digital media spectacle over travel experiences. Tourists transition from freethinking individuals to disciplined subjects, voluntarily investing their leisure time in collective spectacle consumption and the multidimensional imaginaries produced by digital information. The digital media spectacle, shaped by commercial influencer landmarks, becomes a key factor in obscuring the authentic cultural essence of travel.

In shaping tourism through culture and highlighting culture through tourism, the diverse integration of cultural and tourism symbols represents a major trend in the development of China’s cultural industry. Breaking away from the capital-driven allocation of cultural and tourism resources requires tourists to actively enhance their media literacy, break free from digital enclosure, improve their ability to discern digital information, and clarify their own travel needs. Local cultural and tourism departments should leverage algorithmic recommendations while establishing a “Cultural Breeze” pathway to help tourists understand local cultural characteristics and historical connections. By integrating network regulation and algorithm-driven traffic guidance, these initiatives can effectively extend the historical and cultural significance of popular tourist sites, avoiding the fleeting type of tourism boom. By constructing spaces for local interaction and embodied experience, local cultural and tourism departments can optimize the communication pattern of cultural and tourism innovations, thereby enhancing the production of cultural and tourism spaces, shaping the tension of cultural significance, and leading the return of leisure time to individuals’ personal choices.

## 6. Limitations and Future Directions

Although this study includes samples with representativeness in terms of gender and geographic distribution, it lacks a systematic analysis of differences across age groups, occupations, and educational backgrounds. As a result, it falls short of fully capturing the impact of demographic characteristics on media interpretation paths and behavioral choices. Future research could employ structured sampling designs and stratified comparative analyses to better reveal the differentiated effects of media influence across social groups. Moreover, this study primarily analyzes the phenomenon of “group roaming” from a collective perspective, without distinguishing the roles and strategic differences among tourists within group behaviors—such as imitators, initiators, or detached observers. Future studies may draw on behavioral sociology to delve into the internal interaction structures and cognitive positioning within groups. The diversity of platforms and audiences also warrants further attention. While this study covers users of major short-video platforms such as Douyin and WeChat Video Channels, it does not verify the correlational effects between demographic variables (e.g., gender, age) and conformist travel behavior through big data user profiling. How digital media platforms, particularly those targeting specific user groups, amplify emotional resonance and enhance the effectiveness of “digital persuasion” through precise communication remains a question for further investigation. These issues hold both theoretical value and practical significance for future research, especially in informing personalized media recommendation mechanisms and platform cultural strategies.

Despite these limitations, this study offers significant theoretical expansion and empirical support for research on digital media and tourism communication behavior. Drawing from an interdisciplinary perspective that integrates communication studies and social psychology, it combines the “Society of the Spectacle” theory with the “Theory of Planned Behavior” to construct an analytical model of persuasive digital media landscapes and tourists’ conformity-based travel decision-making. This model enhances the depth of research on media effects and improves the explanatory power of behavior prediction. By conceptualizing digital persuasion as a composite process embedded within platform algorithms, visual symbols, and user psychology, this study responds to theoretical concerns about how media in the algorithmic age participates in the disciplining of social behavior. Additionally, it introduces the concept of “group roaming” as a simulated travel phenomenon within digital consumer culture, providing a new lens for examining aesthetic labor, identity performance, and the integration of social behavior in the context of visual media. This perspective also lays the groundwork for shifting tourism communication research from a content–effect analysis to a focus on underlying mechanisms. Building upon this foundation, future research could develop more structured cross-platform comparative frameworks to further enrich the theoretical dimensions of digital media landscape studies.

## Figures and Tables

**Table 1:** An overview of the main interviewees’ information and core viewpoints.

Coding	Gender	Age	Career	Tourist City	Core Viewpoints
ZSP20240415	Female	25	Freelancer	Zibo	Tiktok Heat List recommendations. Take photos and punch cards to share.
LZY20240415	Male	25	Worker	Kaifeng	Affordable price. Want to verify it in person.
CWQ20240417	Male	23	Scenic spot volunteer	Hangzhou	Many people come to livestream. Few individual visitors. Dedicated guidance available.
GLS20240417	Male	24	Master’s degree student	Zibo	Reliving the moments of youth and barbecuing with brothers. Reminiscing about the past.
XAJ20240418	Male	28	Tiktok blogger	Kaifeng	Keeping up with current promotional demands can gain traffic support and attract attention.
ZMY20240423	Male	35	High school teacher	Hangzhou	The internet says that the government and the people have a sense of harmony, and the government and the people are very attractive.
JM20240423	Female	33	Rednote blogger	Hangzhou	The regional flow of traffic drives the need. For work benefits.
LWT20240423	Female	30	Merchant	Zibo	The products are pretty much the same, but businesses that use the internet effectively perform better.
HYF20240423	Male	35	Public servant	Kaifeng	Brainwashed by video all the time. Think it is not too bad to bother choosing.
LTY20240424	Female	48	Public servant	Hangzhou	Know the crowds and do not want to follow suit. However, the family only has time during the holidays.
HSQ20240424	Male	32	Waiter	Zibo	Very different from the national rave online. Ordinary barbecues. Will not come back.
LSY20240424	Male	24	Delivery person	Zibo	Several influencer stores have shut down their delivery platforms outright. Creating queues.
DTT20240425	Female	26	Doctoral student	Kaifeng	Watched the Tokyo Monhualu TV series. Wearing imitation makeup to clock in.
WL20240425	Male	31	Freelancer	Kaifeng	Listen to Li Yugang’s songs and look them up on Tiktok. It feels similar to the video.
DZY20240425	Female	32	Cosmetician	Hangzhou	The first perspective is very inviting to substitute. My favorite blogger shoots a lot.
DD20240425	Female	27	Esports commentary	Kaifeng	Filter in order of likes. Occasionally bad.
SR20240425	Male	28	Host	Hangzhou	The picture does not match the real thing. However, it was sent out anyway. It is all in vain.
ZXG20240426	Female	22	Tiktok blogger	Zibo	The company asked to come. Did not realize much profit. Too many people are shooting.
GGM20240426	Male	30	Athlete	Zibo	The internet says it is useless to eat snacks here. It should be good.
ZMX20240427	Female	24	Master’s degree student	Kaifeng	Come check in at the “Riverside Scene at Qingming Festival”, it is a shame if the photos do not capture its essence. Seeing it with your own eyes etches it deeper into memory.
YY20240427	Female	26	Athlete	Zibo	Visited local friends. Avoided overcrowded spots. Had an amazing experience.
QSY20240428	Male	32	Public servant	Kaifeng	A hidden gem that was usually empty suddenly went viral. Now, even I am an overnight internet sensation.
YCY20240428	Female	21	College student	Hangzhou	Read the Rednote raiders, thought this was the wrong peak. Cannot see the scene at all.
ZZ20240429	Male	25	Volunteer	Hangzhou	Each person holds a camera above their head. Search the internet for pictures.
GSM20240429	Male	34	Tiktok blogger	Zibo	Merchants actively seek cooperation, we are responsible for shooting blockbuster videos.
LJX20240502	Female	23	College student	Kaifeng	Capturing wanderlust in a yearly photo series. Chasing the hottest spots. Guaranteed to turn heads.
YML20240503	Female	38	Freelancer	Hangzhou	Decision fatigue hits hard; every travel video makes me want to pack my bags. Then, I remember: just follow the crowds. Less thinking, more wandering.
NZY20240502	Female	23	College student	Zibo	Camera nomnom first. Must serve looks on social media.
XBX20240409	Male	35	Media company manager	Zibo	The average heat does not last long. Take the initiative to create hot spots to seize the opportunity.
WCY20240430	Female	26	University teacher	Kaifeng	There is comfort in the crowd. Videos of bustling streets make me feel alive.
CS20240501	Female	40	Housewife	Zibo	I read online that there is quite a family. A local internet celebrity said it was good.
JXX20240503	Female	28	Coffee salesperson	Hangzhou	Looking for some peace and quiet. Saw on TikTok that this place was less crowded. Came here and realized I was tricked.
ZQ20240503	Female	30	Tour guide	Zibo	There are rankings and travel guides. People tend to follow video recommendations and data-driven strategies.
LYS20240503	Male	62	Retired worker	Kaifeng	I came back to my hometown and brought my grandson along for a visit; also, to support the local tourism economy.
LPY20240503	Male	22	College student	Kaifeng	I am tired of immersive tourism. Urban roaming videos appeal to me more.
XLJ20240503	Female	27	Freelancer	Hangzhou	Especially like the city walk mentioned in Douyin.

**Table 2:** Tertiary node coding of Chinese tourists’ travel behavior intentions: reference points and coverage rates.

Level 1 Node Encoding	Level 2 Node Encoding	Level 3 Node Encoding	Number of Nodes	Proportion
Tourism Behavioral Intention (1173)	Subjective Norms Regarding Tourism (351)	Influence of Livestreaming	78	18.98%
		Traffic Driven by Short Videos	93	23.91%
		Urban Cultural-Tourism Promotion	85	23.28%
		Official Recommendations	95	23.63%
	Perceived Behavioral Control over Tourism (386)	Perceived Physical and Mental Well-being	102	29.67%
		Checklist-Driven Destination Preference	107	29.86%
		Digital Identity Labeling	83	23.16%
		Repetitive and Persuasive Advertising	94	23.98%
	Attitude Toward Tourism Behavior (436)	Perceived Distance Controllability	105	31.02%
		Perceived Time Flexibility	112	31.02%
		Perceived Budget Affordability	128	33.28%
		Perceived Social and Cultural Value	91	23.86%

## Data Availability

The data are contained within the article.
